# From growth to well-being: pathways and social security effects of coordinating medical resources and population in the Yangtze River Delta region

**DOI:** 10.3389/fpubh.2026.1737816

**Published:** 2026-02-09

**Authors:** Yanmei Tian, Zhuoying Chen, Yitong Su, Shanggang Yin

**Affiliations:** 1 School of Economics and Trade, Shandong Management University, Ji'nan, Shandong, China; 2 College of Geography and Environmental Sciences, Zhejiang Normal University, Jinhua, Zhejiang, China; 3 Zhejiang Key of Digital Intelligence Monitoring and Restoration of Watershed Environment, Zhejiang Normal University, Jinhua, Zhejiang, China

**Keywords:** allometric growth, influencing factors, medical and healthcare resources, permanent resident population, Yangtze River Delta region

## Abstract

The effective allocation of Medical and Healthcare (M&H) resources is crucial to the capacity of the social security system in safeguarding and enhancing residents’ health and well-being. To examine the coordination between this specific social security subsystem and the population it serves within the context of urbanization, this study takes the Yangtze River Delta (YRD) region from 2000 to 2022 as a case study. It focuses on the dynamic relationship between the permanent resident population (PRP) and the supply level of M&H resources. An allometric growth model is constructed to analyze the spatiotemporal evolution of their allometric relationship, and a Boosted Regression Tree (BRT) model is employed to identify the underlying influencing mechanisms. The main findings are as follows: (1) Both the PRP and M&H resources in the YRD region showed a consistent upward trend during the study period, with a spatial pattern generally declining from east to west. (2) In terms of vertical allometric growth, the scale index exhibited an increasing trend, and the allometric relationship evolved through three distinct stages: “PRP expansion,” “basic coordination between PRP and M&H resources,” and “M&H resources expansion.” Regarding horizontal allometric growth, from 2000 to 2010, cities with positive and negative allometric growth were nearly equal in number, displaying a spatial pattern of positive growth in the south and negative growth in the north. In contrast, from 2011 to 2022, most cities experienced positive allometric growth. (3) Factors such as industrial structure, medical consumption, population attraction, economic development, population concentration, and aging were identified as key drivers influencing allometric growth. While the marginal effects of these factors varied, their combined influence facilitated shifts in the allometric relationship. (4) The role of economic mechanisms in driving allometric growth weakened under market forces, whereas social mechanisms played an increasingly significant role. Initially, social factors accelerated growth within a market-regulated context; later, under government macroeconomic regulation, their impact further intensified. Consequently, regional integration in the YRD region progressively extended into the domain of public services, with this process gaining substantial momentum.

## 1 Introduction

The role of social security systems in safeguarding and improving residents’ health and well-being has been brought into sharp focus by a series of large-scale public health crises in the 21st century, including the SARS epidemic, the H1N1 influenza, the Zika epidemic, and the coronavirus disease (COVID-19). A robust social security system, of which the equitable provision of Medical and Healthcare (M&H) resources is a fundamental pillar, is critical for ensuring access to care and sustaining societal progress (1). Against the backdrop of rapid urbanization and population growth, however, regional disparities have emerged in the development of key social sectors, including healthcare. The asymmetric development between the permanent resident population (PRP) and M&H resources can lead to both deficits and wastage in medical supplies, undermining the efficiency of the social security system’s healthcare component and ultimately hindering improvements in public health outcomes.

In 1993, the World Bank Development Report emphasized the importance of providing equitable healthcare services, and in 1996, the World Health Organization (WHO) also emphasized that equity in healthcare services should be demand driven rather than dependent on social privilege (2). To address inequities in global access to health resources, the United Nations adopted the 2030 Agenda for Sustainable Development in 2015, which identified “ensuring healthy lifestyles and promoting well-being for all ages” as the main objective (SDGs3) (3). As the world’s largest developing country, although Chinese medical service capabilities have greatly improved, they are still at a relatively low level globally (4). In 2022, the number of health personnel per thousand people in China will be 10.21, and the number of beds in M&H institutions per thousand people will be 6.92. The problem of regional imbalance in medical resources still exists, especially the lack of high-quality medical resources and uneven distribution of regional medical resources, which have become serious constraints on regional coordinated development and social equity and justice (5, 6). Furthermore, although existing research primarily focuses on the equity of medical resources, few studies have explored the evolutionary stages and threshold effects of the imbalance between the PRP and M&H resources in Yangtze River Delta (YRD) urban agglomeration from the perspective of allometric growth.

This study focused on the configuration and optimization of M&H resources in YRD region. We investigate the evolving relationship between the PRP and the supply level of M&H resources from 2000 to 2022. By construction an allometric growth model, we analyze the spatiotemporal dynamics of this relationship, probing whether the growth of healthcare resources keeps pace with population changes—a key question for assessing the effectiveness of regional social security planning. Furthermore, we employ an enhanced regression tree model to uncover the complex, potentially nonlinear mechanisms driving this allometric growth. Our aim is to provide fresh perspectives on the “population-resource” nexus within urbanization, offering a scientific basis for refining social security policies and fostering coordinated regional development. Ultimately, this research contributes to strategies for achieving more balanced and sustainable urban growth, where the social security system effectively fulfills its role in promoting residents’ health welfare.

## 2 Literature review

The literature review will be conducted from three aspects: the development of allometric growth theory and its application in urban studies, the current state of research on medical and health (M&H) resource supply, and the identified research gaps.

### 2.1 Development and application of allometric growth theory

Allometric growth theory, which originated in biology, has now expanded to various fields including geography, economics, physics, and computer science (7–9). Based on scaling analysis and growth laws, this theory is regarded as one of the universal patterns for understanding the world (10, 11). Geography, particularly urban studies, was among the early fields to adopt this concept. Initial research encountered theoretical dilemmas due to dimensional constraints, however, the introduction of fractal thinking successfully resolved this issue within urban allometric relationships, renewing academic interest in the topic (12, 13). Nowadays, allometric scaling laws are widely applied to the study of complex urban systems, encompassing urban form, ecology, and systems (14, 15). The core of this research lies in investigating the interplay between factors driving urban development and urban scale growth (7, 16–18).

### 2.2 Assessment of spatial disparities in M&H resources

Concurrently, against the backdrop of rapid population aging and evolving lifestyles, the demand for M&H resources is increasing. Assessing whether the current supply can meet this growing demand is crucial for ensuring high-quality medical and health services. Existing research on the spatial disparities of M&H resources primarily unfolds at two levels. At the macro level, studies often use provinces or cities as units, constructing evaluation systems for the accessibility of medical resources using indicators such as the number of medical institutions, healthcare personnel, and hospital beds (2, 19, 20). Methods like factor analysis, the entropy weight method, the Delphi method, and Data Envelopment Analysis (DEA) are employed to assess the overall level and regional disparities (21–24). At the micro level, research focuses on individual cities or specific urban areas. Utilizing Point of Interest (POI) data for medical facilities, studies employ methods such as the two-step floating catchment area method, GIS-based network analysis, and kernel density estimation to conduct in-depth analyses of facility accessibility and spatial service coverage (25–33).

### 2.3 Influencing factors of M&H resources

The supply level of M&H resources is influenced by multiple factors. Among them, the level of economic development, urbanization rate, government financial investment, and human resource management systems are widely recognized as key determinants of resource accessibility and distribution equity (5, 34, 35). Additionally, population distribution and structure, terrain, and spatial accessibility are also considered critical regional factors affecting the supply level of medical resources (36, 37, 70). Regarding analytical methods, existing research primarily involves constructing indicator systems and systematically exploring influences using models such as spatial econometric models, grey relational models, geographical detector, and geographically weighted regression. For instance, Yin et al. (38) selected eight indicators from natural, social, and economic dimensions, using a geographically weighted regression model to reveal the spatially heterogeneous impacts of multidimensional indicators on China’s medical resources. Sun et al. (39) selected multiple indicators from five aspects—economic, medical, educational, social, and other environmental factors—and measured their impact on residents’ medical expenditure by constructing a spatial panel fixed-effects model. Zhou et al. (40) utilized the geographical detector to quantify the single-factor impact intensity and interaction effects of 10 indicators selected from three dimensions—economic strength, social environment, and health investment—on the level of medical resources. Wu et al. (41) employed the grey relational model to analyze the degree of influence of multidimensional indicators encompassing economic, demographic, and social aspects on resource allocation.

### 2.4 Existing research limitations

The review of existing literature shows that while research on the allocation of medical resources has yielded some results, it still faces limitations in three key areas. First, in terms of research scale, most studies focus on macro levels such as the nation or province. They pay relatively little attention to urban agglomerations as an important spatial unit. In particular, there is a lack of theoretical frameworks that treat urban agglomerations as complex systems with internal network connections and scale interactions. This in turn limits the ability to delve into the dynamic mechanisms and cross-scale effects of M&H resources allocation within such regions. Second, regarding research content, existing work mainly analyzes the spatial distribution, allocation efficiency, and influencing factors of M&H resources. However, it often overlooks the fact that the fundamental purpose of allocating such resources is to meet the needs of different population sizes. Establishing the intrinsic link between resource supply levels and urban population scale would provide strong support for research in this field. Finally, methodologically, while spatial statistics or traditional econometric models are commonly used, they tend to ignore the multi-level and nonlinear characteristics of urban agglomerations’ internal structure. This makes it difficult to effectively capture potential allometric growth laws and spatial heterogeneity between medical resources and population size. Moreover, most studies remain at the stage of describing allocation patterns or evaluating static efficiency. Few explore, from a dynamic evolution or mechanism-coupling perspective, the matching logic and theoretical implications between resource supply and urban scale growth during the development of urban agglomerations. As a result, deeper understanding of medical resource distribution patterns is constrained.

## 3 Materials and methodology

### 3.1 Research area and data sources

According to the 2019 YRD Regional Integration Development Plan, the YRD region comprises Shanghai, Jiangsu, Zhejiang, and Anhui. This region encompassed 41 cities at or above the prefecture level, accounting for 3.72% of China’s land area and 16.78% of its population in 2019. Despite its relatively small geographical size, the YRD contributed approximately 24.00% of China’s total economic output. Additionally, the urbanization rate in the YRD region reached 74.30%, well above the national average of 65.22%.

In this study, the urban population was represented by the PRP of each city. The comprehensive index for the supply level of M&H resources was constructed using data on the number of M&H institutions, hospital beds, and doctors within those institutions. The data sources include the Statistical Yearbooks of Various Cities, the China Urban Statistical Yearbooks, and the China Regional Economic Statistics Yearbooks (2001–2023 editions).

To ensure data comparability across cities and over time within the study period (2000–2022), this study applied uniform adjustments to address inconsistencies arising from changes in administrative divisions. The specific procedures are as follows:

First, regarding population data, the “year-end permanent resident population” from the China City Statistical Yearbook and provincial/municipal statistical yearbooks served as the core indicator. In response to administrative changes—such as the conversion of counties into districts or the merger of prefecture-level cities—historical data were retroactively adjusted based on the administrative boundaries as of the end of 2022. The adjustment principle involved merging or splitting historical data for relevant areas to maintain geographical consistency with the final divisions. Data were primarily drawn from detailed county- and district-level tables in the respective statistical yearbooks. For a small number of missing disaggregated figures from earlier years, interpolation or proportional allocation based on higher-level administrative unit totals was applied. Second, for healthcare resources data, figures on the number of medical institutions, hospital beds, and doctors were sourced from the Statistical Yearbooks of Various Cities. The same benchmark adjustment principle used for population data was applied to reconcile changes in statistical units due to administrative restructuring. This ensured that each city’s annual data corresponded to its actual jurisdictional area at the time. Where reporting overlaps or gaps occurred during transitional years, data from the newly established divisions were taken as the reference, and preceding years were merged accordingly. Through these steps, this study aims to construct a panel dataset that maintains consistent comparability across both temporal and cross-sectional dimensions.

### 3.2 Methodology

This study employed a three-step analytical approach. First, a comprehensive index was constructed to measure the supply level of M&H resources. Second, an allometric growth model was developed to analyze the growth relationship between the PRP and the M&H resource supply level in the YRD region. Finally, a Boosted Regression Tree (BRT) model was applied to identify and examine the influencing factors driving this growth. Specifically, the allometric growth model characterizes the relative development between the permanent resident population and M&H resource supply, while the BRT model elucidates the underlying causes and mechanisms of the observed allometric growth patterns. In other words, the allometric model captures the phenomenon and dynamics of the population-resource relationship, whereas the BRT model reveals the mechanisms governing this relationship.

#### 3.2.1 Calculation of M&H resources supply levels

A comprehensive index of the M&H resources supply level was developed based on the quantities of M&H institutions, hospital beds, and doctors (42, 43). To ensure an impartial and data-driven assessment, the entropy method was utilized, allowing for an objective analysis of the information embedded in the data while minimizing subjective biases (44). The range normalization technique was applied to standardize the three variables, after which their respective information entropy and weights were computed. The calculated weights for the numbers of M&H institutions, hospital beds, and doctors were 0.2774, 0.3581, and 0.3645, respectively. These weights were then used in a weighted summation approach to determine the overall index of M&H resources supply in the YRD region, expressed using the following Equation 1:


$$M_{\mathrm{ij}}=\sum_{j=1}^{3}Z_{\mathrm{ij}}\times W_{\mathrm{ij}}$$ (1)

where *M_ij_* represents the supply level of M&H resources in city *i*; *Z_ij_* denotes the standardized value of variable *j* for city *i*, and *w_ij_* is the weight assigned to variable *j*.

#### 3.2.2 Allometric growth model

The concept of “allometric growth” originates from biology and ecology, describing the disproportional growth relationship between parts of an organism and its whole, or among different parts (45). This concept has subsequently been adopted into human geography and regional science to analogize and quantitatively analyze the phenomenon where different elements within a regional system grow at inconsistent rates, along with its spatial implications.

Depending on the analytical perspective, allometric growth can be primarily categorized into two types (46): Vertical allometric growth, which examines the changing relationship between the growth rates of different elements within the same spatial unit over time; and Horizontal allometric growth, which investigates the disparities in the growth rate of a specific element across different spatial units at a given point in time, with the specific model as follows:


$$M_{t}=aP_{t}^{b}$$ (2)

By applying the logarithm to both sides of Equation 2, we obtain the following Equation 3:


$$\ln\;M_{t}=\ln\;a+b\ln\;P_{t}$$ (3)

where *M_t_* and *P_t_* represent the M&H resources supply level and the PRP of the city at time *t*, respectively; *a* and *b* refer to the scale factor and index, respectively. *b* is the allometric growth coefficient.

To classify the growth types, this study uses b = 0.85 as the reference threshold for coordinated growth. This choice is supported by the following considerations:

First, the threshold is well-grounded in both theory and empirical research. Seminal work on urban scaling laws by Bettencourt et al. (47), along with subsequent studies on urban public infrastructure growth by Chen (48) and Lan et al. (49), consistently shows that when the expansion of urban infrastructure—such as roads and utilities—keeps pace with or slightly outpaces population growth under economies of scale, the scaling exponent typically fluctuates around 0.85. This offers a widely recognized quantitative benchmark for identifying a coordinated relationship between resource supply and population size. Second, this threshold is appropriate in the specific context of this research. Our study focuses on M&H resources in cities within the YRD, which are essentially part of urban public infrastructure and services. Although M&H resources have their own professional specificities, their supply is influenced by general urban dynamics such as economies of scale, allocation efficiency, and administrative planning. Adopting the b = 0.85 benchmark—which has been widely validated in related literature—ensures that the classification criteria in this study align with established scholarly frameworks, thereby improving the comparability and interpretability of the findings.

Based on this benchmark, we can examine the direction (positive or negative deviation) and magnitude of each city’s estimated b value relative to 0.85 to effectively reveal spatial disparities and temporal evolution in the allometric growth patterns between M&H resources and population in the YRD urban agglomeration. Table 1 presents the classification and characteristics of the allometric growth types.

**Table 1:** Allometric growth types and characteristics.

Allometric growth type	Classification	Criteria	Relationship	Characteristics
Positive allometric growth (PAG)	PAG level 3	3 ≤ *b*	Strong expansion of M&H resources	The growth rate of the M&H resources supply level significantly exceeds that of the PRP.
	PAG level 2	1 ≤ *b* < 3	Weak expansion of M&H resources	The growth rate of the M&H resources supply level is higher than that of the PRP.
	PAG level 1	0.85 ≤ *b* < 1	Essential alignment of M&H resources with PRP	The growth rate of the M&H resources supply level is slightly higher than that of the PRP.
Negative allometric growth (NAG)	NAG level 1	0.5 ≤ *b* < 0.85	Weak expansion of PRP	The growth rate of the M&H resources supply level is lower than that of the PRP.
	NAG level 2	0 ≤ *b* < 0.5	Strong expansion of PRP	The growth rate of the M&H resources supply level is much lower than that of the PRP.
	NAG level 3	*b* < 0	Shrinkage of M&H resources relative to PRP	Either the M&H resources supply level, the PRP, or both have experienced a decline.

#### 3.2.3 Boosted regression tree model

The BRT model is a self-learning approach that integrates the classification and regression tree algorithm with the boosting technique, enhancing the reliability and stability of the regression tree model (50, 51). The BRT model, which can handle nonlinear relationships, has previously been used in healthcare and related studies on allometric growth (51, 52).

An index system was developed to examine the change in the scale index, using the allometric growth of the PRP and the supply level of M&H resources as dependent variables. The BRT model was employed to establish the relationship between each index and the scale index, and to quantify the connection between these indices and the scale index of both the PRP and M&H resources, while considering other indicators as the average value. The contribution rate of each index to the change in the scale index was then determined. The model was built using the “dismo” R package, with the following parameters: tree complexity = 3, learning rate = 0.01, and all other parameters set to their default values.

## 4 Results

### 4.1 Allometric growth characteristics

#### 4.1.1 Spatial distribution pattern

**Figure 1:**
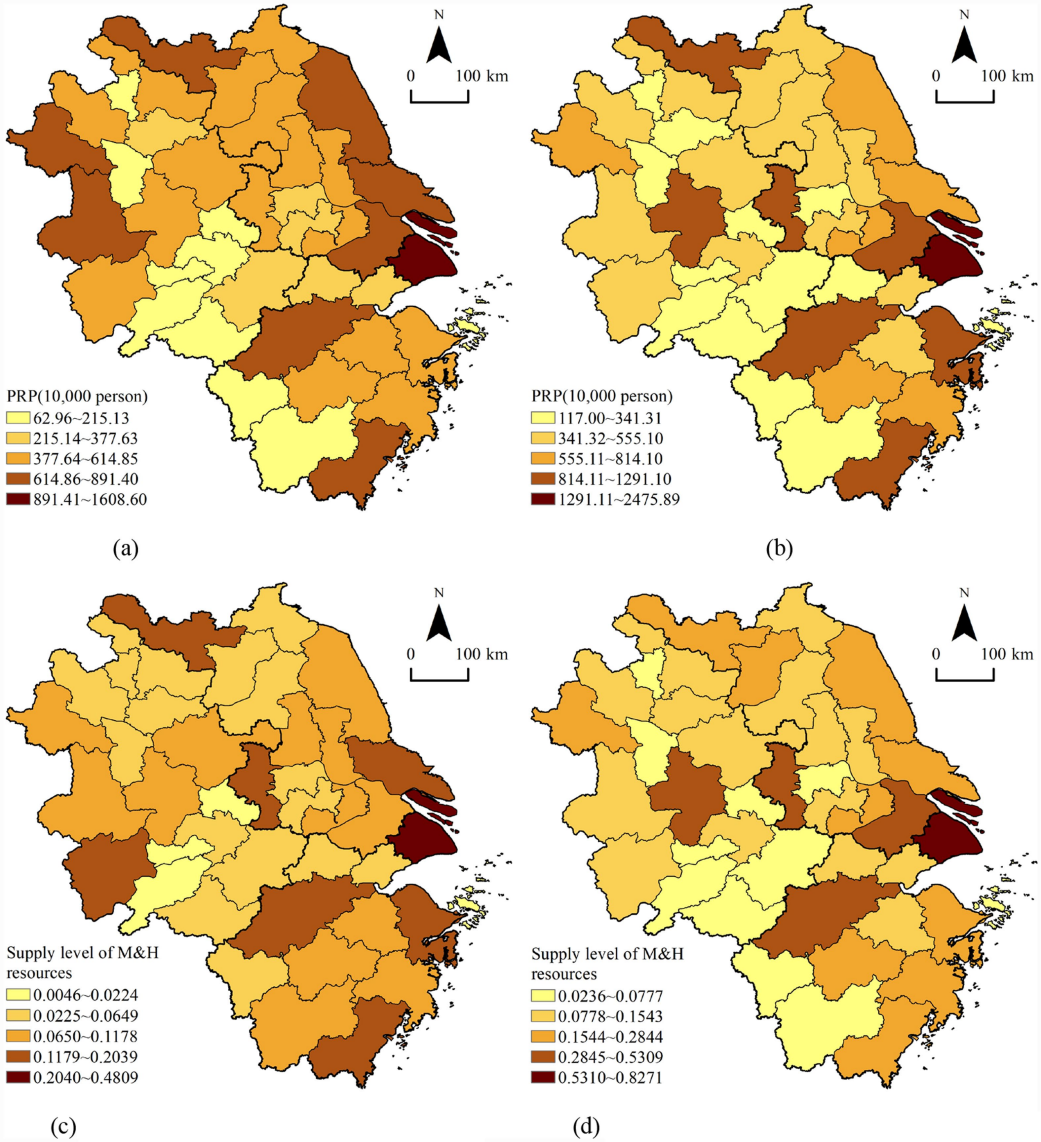
Spatial distribution of the PRP and the supply level of M&H resources in the YRD region in 2000 and 2022. **(a)** PRP in 2000; **(b)** PRP in 2022; **(c)** supply level of M&H resources in 2000; **(d)** supply level of M&H resources in 2022.

We calculated the supply level of M&H resources in YRD cities between 2000 and 2022. The PRP and the supply level of M&H resources were visualized using the ArcGIS software’s natural interruption grading method (Jenks) (see Figure 1).

In terms of the PRP, the spatial distribution remained relatively stable from 2000 to 2022, with high-value areas mainly distributed in Shanghai, eastern Jiangsu, eastern Zhejiang, Suzhou, Hangzhou, Wenzhou, Xuzhou, and other cities in the forefront of the YRD region for a long time. Low-value areas were mainly distributed in southwest Zhejiang, southern Anhui, Lishui, Quzhou, Huangshan, Chizhou, Tongling, Ma’anshan, and other cities where the scale of the PRP had remained low. There were 27 cities (accounting for 65.85% of the study area) with an increase in the PRP scale in the YRD region. They were concentrated in Shanghai, Zhejiang, southern Jiangsu, and central and southern Anhui. Conversely, 14 cities (34.15%) had a reduced size of the PRP and were concentrated in the central and northern Jiangsu as well as the northern and western Anhui.

The spatial pattern changed significantly from 2000 to 2022 with respect to the supply levels of M&H resources. The high-value areas in 2000 were scattered, mainly distributed in Shanghai, Nanjing, Nantong, Xuzhou, Hangzhou, Ningbo, Wenzhou, Anqing, and other cities, whereas the low-value areas were primarily concentrated in southern and northern Anhui and northern Jiangsu. By 2022, the distribution range of high-value areas decreased, mainly in Shanghai, Nanjing, Suzhou, Hangzhou, Hefei, and other cities, whereas the distribution range of low-value areas significantly expanded and was distributed primarily in southwest Zhejiang, southern and northern Anhui, and north-central Jiangsu. In the 37 YRD cities (accounting for 90.24% of the research area), the supply level of M&H resources improved. However, four cities (accounting for 9.80% of the research area) had decreased supply levels of M&H resources. They were mainly concentrated in southern Anhui and southwest Zhejiang.

This comparison suggests that the spatial patterns of the PRP and the supply levels of M&H resources were becoming increasingly similar. That is, their patterns gradually evolved into an overall decreasing trend from the east to the west of the YRD region. In general, with the increase of the scale of the PRP in the YRD region, the supply levels of M&H resources have shown an upward trend.

#### 4.1.2 Vertical allometric growth characteristics

An allometric growth model was constructed to analyze the relationship between the PRP and the supply level of M&H resources. Table 2 presents the relevant parameters of the model. From 2000 to 2022, the scale index ranged between 0.7773 and 1.0604, exhibiting an overall upward trend. Based on the variation characteristics of the scale index, vertical allometric growth was divided into two stages: 2000–2010 and 2011–2022. During 2000–2010, the scale index increased gradually. The sub-period 2000–2003 was marked by weak PRP expansion, with a scale index below 1, suggesting possible challenges such as insufficient investment in medical resources or a lag in healthcare development relative to population-driven demand. In contrast, the years 2004–2010 entered a stage of basic coordination between the PRP and M&H resources, reflecting a dynamic balance between resource allocation and population scale. From 2011 to 2022, the scale index continued to rise overall, climbing from 0.9512 to 1.0319. Basic coordination between population and medical resources was maintained during 2011–2014. The period 2015–2022 signaled a phase of weak M&H resource expansion, with the scale index consistently exceeding 1. This indicates that the overall growth rate of medical resource supply outpaced that of population growth. On one hand, it may reflect the cumulative effects of earlier policies aimed at increasing medical resource investment, thereby enhancing overall supply capacity. On the other hand, it also raises attention to potential future issues such as structural “over-allocation” or inefficiencies in resource utilization.

**Table 2:** Relevant parameters of the allometric growth model.

Year	Scale index	Goodness of fit	Classification of allometric growth
2000	0.7773	0.6847	Negative allometric level 1
2001	0.7913	0.7061	Negative allometric level 1
2002	0.8244	0.7164	Negative allometric level 1
2003	0.8395	0.7436	Negative allometric level 1
2004	0.8501	0.7455	Positive allometric level 1
2005	0.8622	0.7483	Positive allometric level 1
2006	0.8742	0.7655	Positive allometric level 1
2007	0.9004	0.7996	Positive allometric level 1
2008	0.9054	0.8339	Positive allometric level 1
2009	0.9358	0.8634	Positive allometric level 1
2010	0.9395	0.9011	Positive allometric level 1
2011	0.9512	0.9107	Positive allometric level 1
2012	0.9662	0.9334	Positive allometric level 1
2013	0.9747	0.9379	Positive allometric level 1
2014	0.9649	0.9324	Positive allometric level 1
2015	1.0527	0.9458	Positive allometric level 2
2016	1.0524	0.9456	Positive allometric level 2
2017	1.0604	0.9010	Positive allometric level 2
2018	1.0572	0.9026	Positive allometric level 2
2019	1.0541	0.9082	Positive allometric level 2
2020	1.0410	0.9432	Positive allometric level 2
2021	1.0453	0.9406	Positive allometric level 2
2022	1.0319	0.9483	Positive allometric level 2

Between 2000 and 2022, the allometric relationship evolved through three phases: weak PRP expansion, basic coordination between PRP and M&H resources, and weak M&H resource expansion. Over time, particularly after the SARS outbreak in 2003, the supply level of M&H resources continued to improve. The relative growth rate of M&H resources shifted from being lower to higher than that of the PRP. Subsequently, the gap between M&H resource supply and the PRP widened, highlighting the sustained enhancement of M&H resources in the YRD region. Given the regional disparities in M&H resource supply, it is necessary to further examine the horizontal allometric relationship between the PRP and M&H resource supply levels.

From a policy perspective, this evolutionary pathway reflects a phased shift in health resource allocation strategy. The early phase of “weak PRP expansion” revealed a baseline resource shortage, prompting policies aimed at accelerating infrastructure coverage. The transition to and sustainment of the “basic coordination” phase represented a period of effective catch-up and balanced growth, indicating that policy measures successfully aligned medical resource investment with population demand. However, the subsequent entry into the “weak M&H resource expansion” phase carries more complex implications. On one hand, it clearly signifies a notable achievement in the substantial growth of total health resources. On the other hand, it suggests the potential onset of a new developmental stage, in which the central challenge evolves from “how much to invest” to “where to invest and how to optimize investment.” The persistent widening gap calls for a policy shift toward improving spatial equity and allocation efficiency of the expanded resource stock, ensuring that additional investments lead to tangible gains in service quality and accessibility.

#### 4.1.3 Horizontal allometric growth characteristics

**Figure 2:**
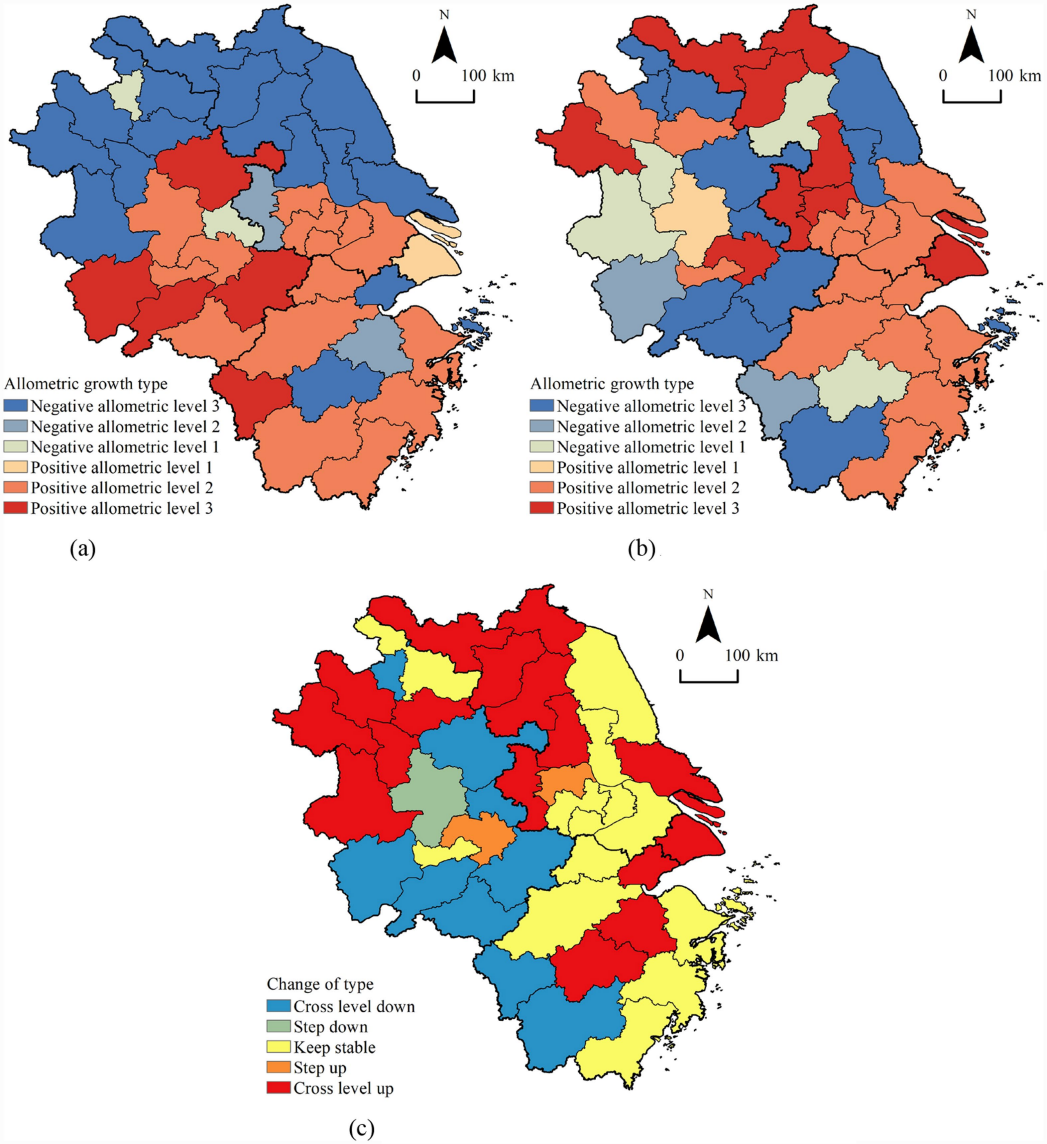
The distribution and change of the allometric growth types in the YRD region. **(a)** Allometric growth types in 2000–2010; **(b)** allometric growth types in 2011–2022; **(c)** change in allometric growth type.

We calculated the fitting model for the allometric growth in each city during 2000–2010 and 2011–2022 and visualized the allometric types and their changes (Figure 2).

Between 2000 and 2010, the YRD region comprised 20 cities exhibiting positive allometric growth and 21 cities showing negative allometric growth. Among these, Shanghai was the only city classified at positive allometric level 1, representing a stage of refined management in which resource growth in a megacity, after reaching high saturation, entered a phase of highly coordinated expansion with population growth. Fourteen cities were identified as positive growth level 2, primarily located in Zhejiang, southern Jiangsu, and central Anhui, while five cities at positive growth level 3 were mainly concentrated in southern Anhui. This spatial pattern can be attributed to: (1) First-mover Economic Advantage: Regions such as southern Jiangsu and northern Zhejiang, driven by export-oriented and private-sector economies, accumulated substantial fiscal capacity, enabling proactive or commensurate investment in healthcare infrastructure. (2) Proactive Regional Policies: Zhejiang province implemented balanced public service system development at an earlier stage, while certain cities in southern Anhui likely benefited from targeted resource allocations due to their positioning within tourism and wellness industries.

On the negative allometric growth side, Ma’anshan and Huaibei were the only two cities at negative allometric level 1; Nanjing and Shaoxing were categorized as negative allometric level 2; and 17 cities fell under negative allometric level 3, mainly distributed across central and northern Jiangsu and northern Anhui. The underlying reasons for this pattern include: during this period, as traditional out-migration sources and economically less developed areas, these regions possessed relatively weak local fiscal capacity, resulting in insufficient sustained investment in the healthcare sector. Simultaneously, rapid urbanization spurred large-scale labor outflow toward core areas of the YRD, leading to a disconnect between statistical growth or structural changes in the PRP and substantive local resource investment, thereby creating a “dilution” effect on resources.

From 2011 to 2022, the number of cities with positive allometric growth in the YRD region was significantly higher than those with negative allometric growth. A total of 24 cities showed positive allometric growth, accounting for 58.54% of the study area. Among them, Hefei was the sole city at positive allometric level 1. Fourteen cities were classified as positive allometric growth level 2, primarily located in southern Jiangsu and northern/eastern Zhejiang, reflecting sustained and robust resource investment. Nine cities were at positive allometric level 3, distributed across Shanghai and northern/western Jiangsu. The potential drivers of this transformation include: (1) Policy-Driven Catch-Up Effect: After 2010, national and provincial strategies such as the equalization of basic public services and “Healthy China” were reinforced. Previously lagging regions like northern Jiangsu received increased fiscal transfers and targeted investments, leading to leapfrog growth in healthcare resources. (2) Core City Radiation and Indigenous Development: Shanghai, as the leading core, contributed to high growth through the expansion of its high-quality medical resources and their outward spillover effects.

Meanwhile, some cities in central and northern Jiangsu entered a phase of accelerated industrialization and urbanization following coastal development and improved transportation infrastructure, driving synchronous resource expansion. Conversely, 17 cities exhibited negative allometric growth, representing 41.46% of the study area. Among these, Huainan, Jinhua, and Lu’an were categorized as negative allometric level 1; Anqing and Quzhou were at negative allometric level 2; and 12 cities were classified as negative allometric level 3, mainly distributed in southern and eastern Anhui. These regions were likely constrained by factors such as topography, economic scale, and fiscal capacity, facing dual challenges in attracting population and sustaining investment in high-cost medical resources, which resulted in persistently sluggish resource growth.

Between the two stages, allometric growth improved in 18 cities (43.90% of the study area). Among them, only Zhenjiang and Wuhu showed improvement over time within the same level, while 16 cities upgraded across levels, demonstrating a clustering trend in northwestern Jiangsu and northwestern Anhui. This visually corroborates the spatial operation of the aforementioned “policy compensation” and “development catch-up” mechanisms. Cities that remained stable were mostly mature areas in southern Jiangsu and northern Zhejiang, where resources and population had already achieved a relatively stable, high-level equilibrium. Ten cities experienced a decline in allometric growth, among which only Hefei declined within the same level, and nine cities downgraded across levels, concentrated in southwestern Zhejiang and southern Anhui. This may be related to more pronounced population outflows in recent years and increased fiscal constraints during local industrial transition in these areas.

Overall, the allometric growth patterns in cities across the YRD region predominantly exhibited positive allometric growth, with the number of such cities steadily increasing over time. In other words, as time progressed, the M&H resources in most cities in the YRD region have shown consistent improvement, with their relative growth rate outpacing that of the PRP. Additionally, the gap between their relative growth rate has been progressively widening.

### 4.2 Analysis of influencing factors of allometric growth

#### 4.2.1 Selection of influencing factors

Drawing on the research and practical work of scholars on the supply levels and allometric growth of M&H resources (9, 40, 53), we identified 10 indicators based on their scientific rigor and data availability. These indicators include the economic development level, industrial structure, fiscal self-sufficiency capacity, medical investment, urban development level, population attraction, population agglomeration, urban–rural income gap, medical consumption, and aging. Incorporating these factors, we constructed a comprehensive framework to analyze their influences on the allometric growth of the PRP and M&H resources supply levels (Table 3).

**Table 3:** Influencing factors of the allometric growth.

Influencing factor	Concrete index	Calculation method	Unit
Economic development level	Per capita GDP	GDP/PRP	Yuan/person
Industrial structure	Proportion of added value of tertiary industry	Added value of tertiary industry/GDP	%
Fiscal self-sufficiency capacity	Ratio of fiscal revenue to expenditure	Local fiscal revenue/local fiscal expenditure	%
Medical investment	Proportion of M&H expenditure in government expenditure	M&H expenditure/local fiscal expenditure	%
Urban development level	Urbanization rate	Urban population/PRP	%
Population attraction	Ratio of PRP to registered population	PRP/registered population	%
Population concentration	Population density	PRP/administrative area	Person/km^2^
Urban–rural income gap	Rural–urban income ratio	Urban income/rural income	%
Medical consumption	M&H expenditure as a share of consumer spending	M&H expenditures/living expenses	%
Aging	Proportion of people aged 65 and older	People aged 65 and older/total population	%

The selection of various influencing factors is based on the following reasoning: On one hand, the income level of residents is closely tied to the overall economic development. As the economy develops, residents’ income rises, thereby increasing the demand for high-quality M&H services (54). Additionally, the optimization of the industrial structure, especially the growth of high-tech and service industries, introduces new technologies, equipment, and service models to the M&H sector, contributing to the high-quality development of the sector. On the other hand, these two factors reflect the economic strength of a city, which is crucial for attracting external populations and improving urban M&H resources. In this sense, they serve as a material foundation for the enhancement of M&H services in urban areas (55).

Second, in terms of administration, we measure fiscal self-sufficiency capacity, medical investment, and urban development levels. Fiscal self-sufficiency capacity determines the ability of local governments to invest in M&H services (56). The level of medical investment directly affects the health and quality of life of urban residents, while the level of urban development is a key factor shaping both fiscal self-sufficiency and medical investment. All three factors are strongly linked to administrative capacity and form the foundation for developing urban M&H resources. They are also critical to the broader economic development of a city. Together, these factors provide a crucial dimension for evaluating the supply level of M&H resources in any given city or region.

Third, in terms of population, we focus on population attraction and concentration levels. Cities with high levels of population attraction typically exhibit stronger economic vitality, more employment opportunities, and a higher quality of life. These factors draw a significant influx of external populations (57), thereby increasing the demand for M&H services. Population concentration, on the other hand, reflects the density of the population in a region, directly affecting the allocation of M&H resources (4). Both population attraction and concentration levels are indicators of a city’s appeal and demographic distribution, which in turn shape the demand for and supply of M&H resources.

Finally, in terms of society, we consider the urban–rural income gap, medical consumption, and aging. The urban–rural income gap affects the allocation of M&H resources (58), reflecting disparities in urban–rural development. Medical consumption level indicates residents’ spending on healthcare, which not only reflects their health needs but also signals the health burden of society. Aging is a key indicator of the social burden, as it highlights the proportion of older adults in the population and the corresponding demands on M&H services (59). Together, these factors—income disparities, medical consumption, and aging—impact the development of M&H resources and the flow of populations.

#### 4.2.2 Factor contribution rate

The BRT model was employed to analyze how different factors influenced the allometric growth of the PRP and the supply level of M&H resources in the YRD region during two periods: 2000 to 2010 and 2011 to 2022 (Table 4).

**Table 4:** Contribution rate and its ranking of each factor to allometric growth.

Influencing factor	2000–2010		2011–2022	
	Contribution rate (%)	Contribution ranking	Contribution rate (%)	Contribution ranking
Economic development level	9.94	3	12.16	3
Industrial structure	28.82	1	11.11	5
Fiscal self-sufficiency capacity	2.21	9	5.06	8
Medical investment	1.78	10	10.34	6
Urban development level	7.51	5	1.81	10
Population attraction	26.02	2	1.83	9
Population concentration	5.98	6	15.44	2
Urban–rural income gap	3.41	8	5.73	7
Medical consumption	5.40	7	25.01	1
Aging	8.93	4	11.51	4

In the period from 2000 to 2010, the industrial structure and population attraction were the most influential factors on allometric growth, each contributing over 25%, followed by economic development and aging, with a contribution rate exceeding 8%. Factors such as urban development level, population concentration, medical consumption, the urban–rural income gap, fiscal self-sufficiency capacity, and medical investment had relatively low impact, each contributing less than 8%. In the period from 2011 to 2022, medical consumption had the greatest effect on allometric growth, with a contribution rate of 25.01%, followed by population concentration, economic development, aging, industrial structure, and medical investment, all contributing over 10%. Other factors—such as the urban–rural income gap, medical consumption, medical investment, economic development, population attraction, fiscal self-sufficiency, and urban development—exerted a weaker influence, with each contributing less than 10%.

Our analysis of the contribution rates of various factors between 2000–2010 and 2011–2022 revealed an upward trend in the influence of several factors, including economic development, fiscal self-sufficiency, medical investment, population concentration, the urban–rural income gap, medical consumption, and the aging. Notably, the contribution rates of medical consumption and population concentration surged to become the top two factors, significantly enhancing their impact on allometric growth. Additionally, the contribution rates of fiscal self-sufficiency, medical investment, and the urban–rural income gap also increased, leading to improved rankings for these factors to varying extents.

Additionally, the contribution rates of industrial structure, urban development, and population attraction to allometric growth showed a downward trend. Specifically, the contribution rates of industrial structure and population attraction decreased by more than 15%, with their impact on allometric growth dropping from the first and second positions to the fifth and ninth positions, respectively. Similarly, both the contribution rate and ranking of urban development also declined.

#### 4.2.3 Influence of leading factors

**Figure 3:**
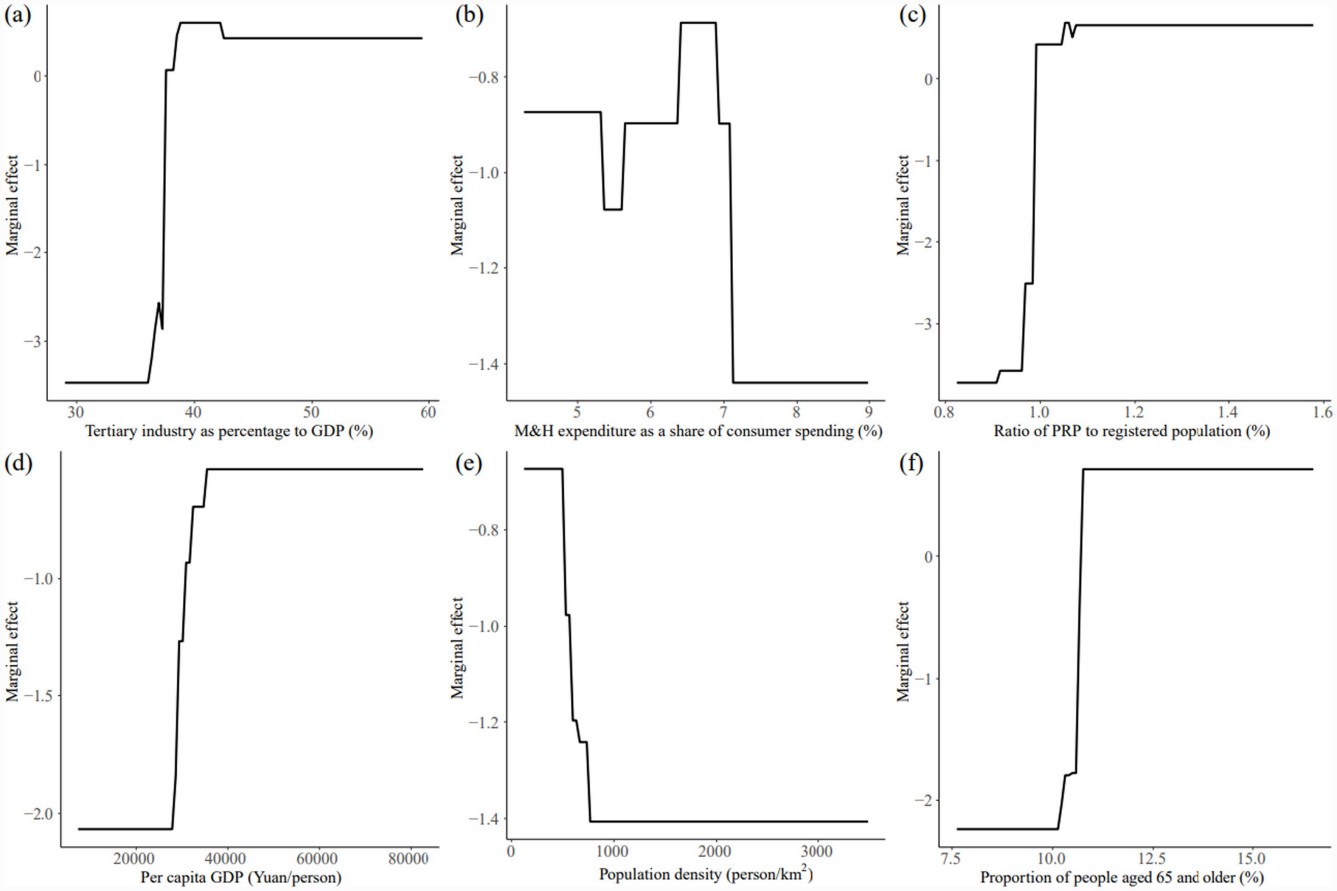
Marginal effects of dominant factors on allometric growth during 2000–2010: **(a)** Industrial structure, **(b)** medical consumption, **(c)** population attraction, **(d)** economic development level, **(e)** population concentration, and **(f)** aging.

**Figure 4:**
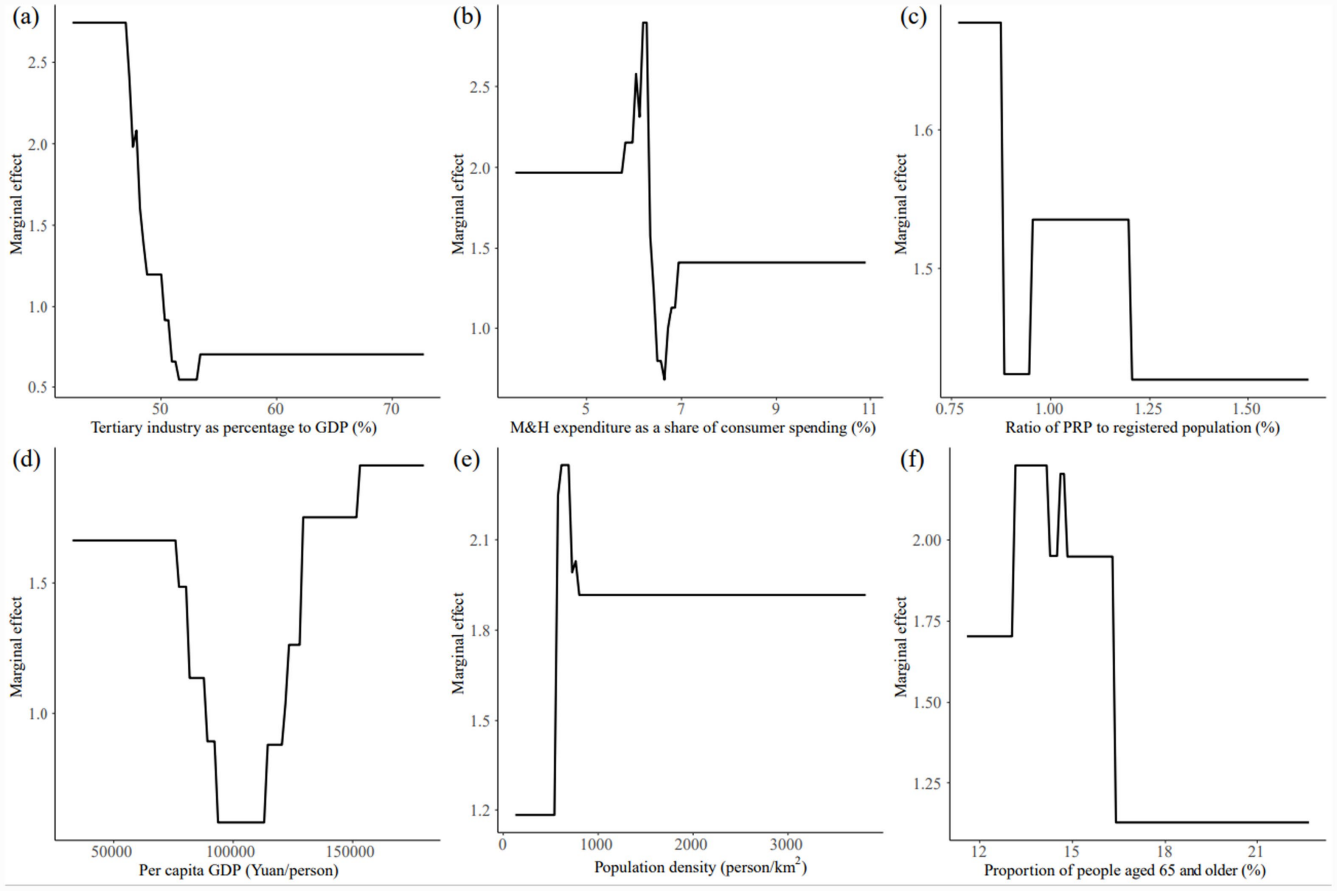
Marginal effects of dominant factors on allometric growth during 2011–2022: **(a)** industrial structure, **(b)** medical consumption, **(c)** population attraction, **(d)** economic development level, **(e)** population concentration, and **(f)** aging.

We calculated the average contribution rates of each factor for the two periods, 2000–2010 and 2011–2022, and identified the top six factors as the leading factors. In descending order, these were: industrial structure, medical consumption, population attraction, economic development, population concentration, and aging. The average contribution rate of these leading factors was above 10%, indicating that they had a significant impact on the allometric growth of the urban population and the supply levels of M&H resources in the YRD region (Figures 3, 4).

1. The influence was primarily driven by economic factors, with economic development level and industrial structure serving as the key drivers. The marginal effects of economic development level on allometric growth shifted from negative to positive, showing an overall positive correlation. As per capita GDP increased, residents’ income rose, leading to greater demand for high-quality M&H resources. Consequently, the impact of economic development on allometric growth generally strengthened over time. In contrast, the marginal effect of industrial structure on allometric growth was negative in the early stage and became positive later, shifting from a positive correlation to a negative correlation. The intensity of its impact on allometric growth decreased significantly, mainly because as the proportion of the tertiary industry grew, both the urban population and M&H resources supply level increased. However, as the proportion of the tertiary industry stabilized in the later stage, its influence on population growth and healthcare began to decline. This shift reflects the initial growth driven by the expansion of the tertiary sector, followed by a reduction in its impact once the sector reached a stable share of the economy.
2. The influence was primarily driven by demographic factors, with population attraction and population concentration being the key drivers. The marginal effect of population attraction on allometric growth shifted from negative to positive, showing a positive correlation in the early stage and an overall negative correlation in the later stage. Overall, the marginal effect increased, and population attraction level reflected the city’s ability to attract foreign population. Despite this, the marginal effect increased over time, reflecting the city’s growing ability to attract external populations. In the early stages, population attraction directly influenced the increase or decrease of the urban population, thereby enhancing its impact on allometric growth. However, as the urban population structure evolved due to population attraction, it subsequently boosted the supply of M&H resources, significantly improving its effect on allometric growth compared to the earlier stage. The marginal effect of population concentration on allometric growth shifted from negative to positive, with the negative correlation turning into a positive one. As population density increased, urban populations grew, and the supply of urban M&H resources improved. However, this effect showed a certain lag. Over time, as population density continued to rise, its influence on allometric growth intensified.
3. The influence was primarily driven by social factors, with medical consumption and aging serving as the key driving forces. The marginal effect of medical consumption on allometric growth shifted from negative to positive, though overall, it exhibited a fluctuating downward trend. This was largely due to the continuous improvement in medical consumption driven by economic development and rising household incomes. However, in the context of promoting the equalization of public M&H services in China, a supply-driven resources allocation model was implemented. As a result, the uneven distribution of resources diminished the impact of improvements in medical consumption on the allometric growth of M&H resources supply. Meanwhile, the marginal effect of the aging on allometric growth gradually shifted from negative to positive. Initially, the aging and its impact on allometric growth were relatively low, but as the older population continued to grow, so did their demand for M&H resources, which significantly influenced allometric growth.

## 5 Discussion

### 5.1 Influence mechanisms of allometric growth

The spatio-temporal evolution of the PRP and the supply level of M&H resources in the YRD region is shaped by the interplay of economic and social mechanisms, which in turn influence the allometric growth between these variables. These mechanisms are interrelated and mutually reinforcing.

The economic mechanism encompasses factors such as economic development, industrial structure, and population attraction and concentration. Moreover, the composition of influence within the economic mechanism has undergone notable changes. In the earlier stages, extensive economic factors—represented by industrial restructuring and population attractiveness—played a dominant role. As development progressed, the contribution of these factors diminished. In contrast, the influence of the level of economic development remained stable or even increased slightly, while that of population concentration rose significantly. This indicates that the economic mechanism has not simply weakened, but has shifted from relying on extensive drivers—such as industrial transformation and in-migration—to leveraging intensive drivers anchored in the existing economic base and the agglomeration effects within urban space. This transition aligns with the general pattern observed in the mid-to-late stages of urbanization. The social mechanism includes medical consumption and population aging. The impact of medical consumption grew over time, while the effect of aging remained relatively stable. This suggests that social mechanisms exert a strong and increasingly important influence on allometric growth.

The improvement in economic development and industrial structure will foster higher levels of population attraction and concentration. Changes in the population, in turn, positively influence economic development and industrial structure. These four factors collectively contribute to the growth of the PRP and generate an increased demand for M&H resources. Rising levels of aging, meanwhile, drive the construction of M&H facilities, thereby enhancing the provision of M&H care. As medical consumption improved, the demand for M&H services among residents grew, which in turn boosted the supply of M&H resources. This dynamic affects the allometric relationship between the PRP and the supply of M&H resources. When the growth rate of the PRP outpaces the rate of improvement in M&H resources—i.e., when the relative growth rate of the PRP exceeds that of the M&H resources, or when the number of PRP increases while the level of M&H resources declines—the coefficient of allometric growth tends to decrease. Conversely, when the rate of improvement in the medical level surpasses that of the PRP—i.e., when the relative growth rate of M&H resources exceeds that of the PRP, or when the M&H resources improve while the PRP decreases—the coefficient of allometric growth tends to increase.

The economic mechanism is primarily driven by market forces, and its impact on allometric growth is mainly reflected in changes in urban population size. As such, its influence is more direct, although the intensity of this impact tends to weaken over time. In the early stages, the social mechanism is also largely shaped by market dynamics; however, in the later stages, government macro-regulation plays a more significant role. The social mechanism’s impact on allometric growth is often channeled through the economic administrative framework. Various factors, such as economic development, industrial structure, population attraction, and medical consumption, bear the influence of market mechanisms, while population concentration and aging reflect the impact of social mechanisms. Importantly, the effects of these factors on the allometric growth rate are not independent; rather, their interactions and synergies can significantly shape the overall growth dynamics. Therefore, it is crucial to consider the interdependence and interaction between economic and social mechanisms when studying the allometric growth of the PRP and M&H resources in a region.

### 5.2 Impact of public health crisis on allometric growth

In recent years, the frequent occurrence of international public health emergencies has significantly impacted the global economy and placed immense pressure on healthcare systems worldwide. The SARS epidemic of 2003, in particular, highlighted long-neglected public health issues in China and prompted reflection by the government, society, and scholars on the allocation of public healthcare resources (60). This crisis also spurred regions to increase their investment in M&H resources. The ongoing research on the PRP and the supply levels of M&H resources in the YRD region supports this observation. According to the 2020 seventh population census, 18.7% of China’s population was aged 60 years and above, with 13.5% aged 65 and older. This demographic shift indicates that China has entered an aging society (61) and is moving towards a “moderately aging” society, which presents additional challenges to the healthcare system and health security infrastructure.

Public health crisis, such as the SARS epidemic in 2003 and the COVID-19 epidemic in 2020, have significantly influenced the relationship between the PRP and the supply level of M&H resources. From 2000 to 2003, this relationship was characterized by a basic coordination between population growth and the availability of medical resources. However, following the SARS epidemic in 2003, cities across China strengthened the construction of M&H resources, and the government focused on achieving a more balanced layout of M&H resources to enhance regional healthcare capabilities. As a result, the allometric growth relationship shifted into a phase of weak M&H resources expansion starting in 2004. This is consistent with findings by (62), who observed that provincial M&H resources in China were growing rapidly and regional disparities were narrowing. From 2020 to 2022, although the allometric growth relationship remained in the weak stage of M&H resources expansion, the allometric growth coefficient showed a significant decline, indicating a slowdown in the growth rate of M&H resources. One reason for this is that, in response to the COVID-19 pandemic, cities at all levels began prioritizing improvements in M&H resources, with a particular focus on building high-level and specialized hospitals. This shift marked a transition from the expansion of M&H resources to an emphasis on quality improvement. On the other hand, as China’s urbanization process has entered a phase of transformation and upgrading, the growth rate of PRP in major cities has gradually stabilized, and the demand for M&H resources has become increasingly saturated. Meng et al. (35) found that the efficiency of M&H resources allocation in Chinese township health centers declined from 2012 to 2021, primarily due to the unchecked expansion of large urban hospitals, with the growth rate of beds in urban medical institutions outpacing that in township health centers. This finding aligns with the conclusion of the present study, which identifies the period from 2004 to 2022 as a phase of medical resources expansion.

To enhance the affordability and accessibility of M&H services, the Chinese government has introduced a series of effective healthcare reforms. The SARS outbreak in 2003 and the widespread issue of “difficult and expensive medical treatment” prompted both government and society to rethink market-driven M&H policies, leading to a renewed focus on public welfare in M&H services. This shift has contributed to increased population mobility between urban and rural areas, along with a growing urban PRP. As a result, both the PRP and the supply level of M&H resources have expanded rapidly. In 2009, China officially launched a new healthcare reform, emphasizing the public welfare nature of healthcare and the leading role of government. By 2015, the government introduced the “Healthy China” initiative, aiming to build a national public health service system. Consequently, after 2015, the allometric growth in the YRD region entered a phase where M&H resources expansion slowed, but the supply of M&H resources saw significant improvements. In their study, Tao et al. (63) summarized the achievements and experiences of China’s health insurance system, drug supply and health system, medical service system, and public health service system since the implementation of the new healthcare reform. They also pointed out that the development of China’s M&H resources supply level has made steady progress as a result, which also confirms this discussion result.

### 5.3 Development path under allometric growth

During the process of urbanization, the expanding PRP in cities has led to long-standing shortages in basic public services such as healthcare, public transportation, and housing, as noted in several studies (64, 65, 69). The report of the 19th National Congress of the Communist Party of China also acknowledged that the primary societal conflict in China has shifted to an imbalance between the people’s growing desire for a better life and unbalanced and insufficient development. This imbalance is particularly evident in the uncoordinated growth of the PRP and the supply of M&H resources, as well as in regional development disparities. As China transitions from high-speed growth to high-quality development, the relationship between the PRP and the availability of M&H resources will continue to shape the trajectory of regional urbanization, ultimately influencing the quality of development in various regions.

The PRP and the supply of M&H resources are both crucial elements of population urbanization and social urbanization, and they should be prioritized equally. In the YRD region, the growth rate of M&H resources supply has outpaced that of the PRP. With reference to the orderly flow and equal exchange of social, economic and environmental factors between urban and rural areas mentioned by Ma et al. (66) and Shan et al. (67) in their respective studies, reasonable allocation of resources between urban and rural areas can be realized, and the improvement of employment policies and social security systems can effectively promote urbanization. The household registration system is an important factor hindering the development of population urbanization. In order to promote population urbanization, we propose to take the following measures: First, promote urban–rural integration and facilitate the transformation of rural residents into urban citizens. Improving the quality of life for rural populations can boost their income levels and help narrow the gap between urban and rural areas. Second, deepen the synchronized reform of the household registration system, employment policies, social security, and other key systems. Such reforms can enhance the urbanization rate, encourage population mobility, and create favorable conditions for equal access to public services for migrant populations. Finally, strengthen fertility-supporting policies by aligning them with relevant economic and social policies. This can be achieved by improving tax, housing, and other supportive measures, which would promote the long-term, balanced development of the population.

To enhance the supply of M&H resources, the focus must shift from “quantity accumulation” to “quality improvement.” This can be achieved through the following measures: First, it is essential to strengthen the service capacity of grassroots medical institutions and optimize the allocation of human resources within the healthcare sector. Additionally, improving systems for the prevention, control, and treatment of major epidemics, as well as enhancing emergency response mechanisms for public health crises, will strengthen overall healthcare resilience. Second, the allocation structure of resources should be optimized to promote a more balanced distribution of high-quality M&H resources. Special attention should be given to the health of the older population, promoting healthy and active aging, and improving the level of medical security for older adults. Finally, efforts should be made to enhance the integration of M&H resources, with key support directed to regions like southwest Zhejiang and southern Anhui, where the M&H resources supply is currently lower. Strengthening interregional cooperation and negotiation in the M&H sector can also play a vital role in addressing regional disparities and improving resource allocation. As proposed by Yuan et al. (68), China needs to continuously expand the quantity of high-quality healthcare resources, including strengthening financial investment sources in the healthcare sector, encouraging social capital support and participation in healthcare, guiding social forces to improve medical facilities and equipment, and a series of measures to address the problem of “insufficient” development of M&H resources supply.

### 5.4 Advantages, limitation and future directions

Building upon the existing limitations in urban agglomeration-scale and allometric growth analysis, this study makes contributions primarily at the following two levels. Specifically, methodologically, by nesting an allometric growth model within a BRT framework, we move beyond the linear or static models commonly used in prior research to characterize the relationship between M&H resources and population size. This design not only dynamically identifies the phased transitions and scale features of M&H resources-population allometric growth within urban agglomerations, but also further disentangles the marginal contributions and threshold effects of driving factors such as industrial structure, M&H consumption, and population aging. In doing so, it addresses the shortcomings of traditional coordination-index models, which often treat underlying mechanisms as a “black box” with insufficient explanation. From an analytical perspective, this study views the “urban agglomeration” as a complex system characterized by intrinsic network linkages and scale interactions. It explicitly incorporates the dual regulatory mechanisms of the market and the state into the full-process analysis of allometric growth, revealing an evolutionary pattern in which the role of economic mechanisms diminishes while social mechanisms progressively strengthen under conditions of regional integration and macro-level governance. Consequently, this research not only operationalizes the policy ideal of equalized public services into a measurable “spatially dynamic” process, but also provides a reference toolkit for analyzing population-resource coordination in the YRD and other regions experiencing rapid urbanization. Furthermore, it offers a precise policy foundation for the tiered allocation of M&H resources, cross-regional integration of public services, and the refinement of social security systems.

Urbanization offers a broad platform for both urban and rural residents to benefit from the fruits of economic and social development, while also fostering more equitable access to M&H services across different regions and social groups. It is anticipated that the integration of public services, particularly in the M&H sector, in the YRD region will continue to accelerate, with population growth and social urbanization becoming increasingly coordinated. This will inject new vitality into the process of regional integration. The high-quality development of the YRD region can provide theoretical basis and practical experience for promoting the sustainable development and prosperity of the Yangtze River Economic Belt and even the entire Chinese society, and for coordinating the development of population and M&H resources. This article is based on the study of the allometric growth of PRP and M&H resources supply level, and deeply explores its spatio-temporal changes and influencing factors in the YRD region. However, there are still some shortcomings in this study: firstly, although the factors influencing positive and negative allometric growth were identified, a systematic comparison of the differences in their underlying driving mechanisms was not conducted. This may render the proposed policy recommendations less effective and less targeted in addressing the specific issues faced by regions at different development stages or exhibiting different allometric types. Secondly, only a single administrative level city, namely the 41 prefecture level cities in the YRD, was analyzed and compared, failing to cover more administrative levels and geographical ranges. This study only explored the relationship between PRP and supply levels of M&H resources, without delving into the interrelationships between different population types and categories of M&H resources.

Based on the above findings and limitations, future research can be further deepened in the following specific directions. First, in terms of analytical scale, future studies could adopt micro-level data at the county or even township and street level. This is not merely a refinement of spatial scope, but an extension to questions not fully addressed in this paper: Does the allometric growth relationship within urban agglomerations exhibit significant hierarchical differences and scale-dependent effects? To this end, by constructing panel models that incorporate spatial and hierarchical interaction terms, or by conducting tests for coefficient differences across subsamples at different development stages, the methodological approach can move beyond overall stage division toward a more dialectical mechanism comparison and validation. Second, regarding the depth of mechanisms, future research could shift from the current aggregate “population-resource” matching toward a structural supply–demand analysis of “sub-populations and categorized resources.” This would address the allocation logic of specific population groups and resource categories, which could not be fully explored in this study due to length constraints. This would require further integration of micro-level survey data and facility-usage data, employing methods such as system dynamics or agent-based modeling to construct complex system scenarios involving “population mobility–resource allocation policies–regional development.” Doing so would allow testing the long-term effects of different regional policies on the balanced allocation of M&H resources through dynamic simulation, thereby addressing this study’s limitations concerning long-term policy analysis. These efforts would not only help explain the heterogeneous mechanisms of resource allocation in urban agglomerations at a finer scale, but would also promote the application and expansion of allometric growth theory within public service research under a more systematic framework. This would in turn provide more forward-looking support for improving cross-level governance and social security systems in regions such as the YRD.

## 6 Conclusions and policy implications

### 6.1 Conclusion

This paper examines the relationship between the scale of the PRP and the supply levels of M&H resources in 41 cities in the YRD region from 2000 to 2022. The study explores the spatial–temporal evolution and the influencing factors of this relationship. The main conclusions are as follows:

1. The PRP and the supply levels of M&H resources in the YRD region exhibited a decreasing spatial trend from east to west. Their spatial distribution followed a similar pattern of gradual increase, with absolute values showing an upward trend over time. In terms of vertical allometric growth, the scale index generally trended upward, and the allometric relationship evolved through three stages: “PRP expansion,” “basic coordination between PRP and M&H resources” and “M&H resources expansion.” Regarding horizontal allometric growth, between 2000 and 2010, the number of cities experiencing positive and negative allometric growth was roughly equal, with a distribution pattern of negative allometric growth in the north and positive allometric growth in the south. From 2011 to 2022, cities with positive allometric growth predominated across the region.
2. The industrial structure, medical consumption, population attraction, economic development, population concentration, and aging were the key factors influencing allometric growth. Among these, the impacts of population concentration, medical consumption, and aging increased over time, while the effects of the other factors diminished. Notably, the marginal effects of industrial structure, population attraction, and aging on allometric growth shifted from a positive to a negative correlation. In contrast, the marginal effect of population concentration changed from negative to positive. The marginal effect of economic development generally exhibited a positive correlation with allometric growth, while the marginal effect of medical consumption showed a fluctuating downward trend.
3. The allometric growth of the PRP and the M&H resources supply in the YRD region were shaped by both economic and social mechanisms. The influence of the economic mechanism on allometric growth gradually diminished due to market regulation, while the social mechanism grew increasingly significant, driven by its dual role in both the market and government. These economic and social mechanisms became integrated, complementing each other toward a common goal.

### 6.2 Policy implications

Building on this core finding, this study proposes more targeted and actionable policy implications to foster deeper integration of regional public services:

First, implementing differentiated resource allocation strategies with categorized guidance. Policymaking must fully consider the spatiotemporal disparities in allometric growth. For city clusters exhibiting negative allometric growth, especially NAG-level 2 and 3 types, parts of southern Anhui and southwestern Zhejiang, the policy focus should be on filling shortages and preventing dilution. Provincial governments should enhance the precision of fiscal transfers and specialized planning, prioritizing the development of basic medical facilities and staffing in these areas, ensuring resource accessibility in population-concentrated zones, and curbing the decline of per capita resources. For cities with positive allometric growth, especially PAG-level 3 types, the policy emphasis should shift to optimizing structure and improving efficiency, guiding them to focus on enhancing service quality and optimizing system architecture alongside aggregate resource expansion, thereby avoiding redundant construction and idle capacity.

Second, establishing a population dynamics centered planning and data platform. To achieve intensive driving, it is recommended that provincial health and natural resource authorities take the lead in jointly establishing a dynamic “population-resources-economy” monitoring and early-warning platform. This platform should integrate real-time data on population mobility and resource utilization efficiency. Key indicators such as permanent resident density and population attractiveness index should be formally incorporated into the core calculation framework of regional health planning. This will shift resource allocation from static planning based on registered populations to dynamic service management oriented to the PRP, thereby responding more sensitively to new demands arising from intra-urban spatial restructuring.

Finally, deepening the integration of social mechanisms with market and government actions. In response to social trends like aging and upgrading medical consumption, policies should design composite intervention tools. For instance, local governments can partner with social capital to develop integrated older adults care and medical service networks in city clusters with high aging degrees. Utilizing the leverage of regional integrated healthcare payment system reforms can guide the rational flow of medical consumption and encourage the diffusion of high-quality resources from core cities to surrounding areas through technical collaboration and telemedicine. This approach aims to meet social needs while stimulating market vitality and alleviating the pressure on sole government provision.

## Data Availability

The raw data supporting the conclusions of this article will be made available by the authors, without undue reservation.
